# Volunteering With Mild Cognitive Impairment: Implications for Subsequent Cognitive Changes

**DOI:** 10.1093/geroni/igaf122.563

**Published:** 2025-12-31

**Authors:** Meng Huo, Kyungmin Kim

**Affiliations:** University of California, Davis, Davis, California, United States; Seoul National University, Seoul, Republic of Korea

## Abstract

Volunteering has cognitive benefits in later life and has been theorized to protect against Alzheimer’s disease and related dementias (ADRD). A small but growing body of volunteering programs target people with mild cognitive impairment (MCI)—who are presumably at elevated risk for ADRD, but we know surprisingly little about who volunteers in the presence of MCI and how volunteering affects their subsequent cognitive changes. The current study sought to address these gaps in the literature. We used longitudinal data from the Health and Retirement Study (2002–2018) and identified a pooled sample of 6,930 midlife and older adults (aged 50+) who met criteria for MCI based on their cognitive scores on the Telephone Interview for Cognitive Status (TICS). We tracked these participants’ sociodemographic characteristics, volunteer activities, and cognitive scores in the subsequent four years. A two-level logistic regression showed that among midlife and older adults with MCI, those who were younger, attained more years of education, had greater wealth, reported better self-rated health, had fewer functional limitations, and reported a history of volunteering were more likely to report volunteering in the presence of MCI. Volunteers with MCI—particularly those who continuously volunteered or initiated volunteering exhibited more positive cognitive changes over time. This study highlights the importance of socioeconomic resources and health in predicting volunteering with MCI and reveals lasting cognitive benefits of volunteering as midlife and older adults adjust to their cognitive impairment. Findings call for more tailored volunteering opportunities for people with MCI.

